# N‐homocysteinylation of DJ‐1 promotes neurodegeneration in Parkinson's disease

**DOI:** 10.1111/acel.14124

**Published:** 2024-02-21

**Authors:** Tao Guo, Lingyan Zhou, Min Xiong, Jing Xiong, Juan Huang, Yiming Li, Guoxin Zhang, Guiqin Chen, Zhi‐Hao Wang, Tingting Xiao, Dan Hu, Anyu Bao, Zhentao Zhang

**Affiliations:** ^1^ Department of Neurology Renmin Hospital of Wuhan University Wuhan China; ^2^ Department of Neurology Second Affiliated Hospital of Nanchang University Nanchang China; ^3^ Department of Clinical Laboratory Renmin Hospital of Wuhan University Wuhan China; ^4^ TaiKang Center for Life and Medical Sciences Wuhan University Wuhan China

**Keywords:** dopaminergic neurons, homocysteine, mitochondrion, oxidative stress, Park7

## Abstract

DJ‐1, also known as Parkinson's disease protein 7 (Park7), is a multifunctional protein that regulates oxidative stress and mitochondrial function. Dysfunction of DJ‐1 is implicated in the pathogenesis of Parkinson's disease (PD). Hyperhomocysteinemia is associated with an increased risk of PD. Here we show that homocysteine thiolactone (HTL), a reactive thioester of homocysteine (Hcy), covalently modifies DJ‐1 on the lysine 182 (K182) residue in an age‐dependent manner. The N‐homocysteinylation (N‐hcy) of DJ‐1 abolishes its neuroprotective effect against oxidative stress and mitochondrial dysfunction, exacerbating cell toxicity. Blocking the N‐hcy of DJ‐1 restores its protective effect. These results indicate that the N‐hcy of DJ‐1 abolishes its neuroprotective effect and promotes the progression of PD. Inhibiting the N‐hcy of DJ‐1 may exert neuroprotective effect against PD.

## Graphical abstract

N‐homocysteinylation of DJ‐1 by HTL promotes neurodegeneration.

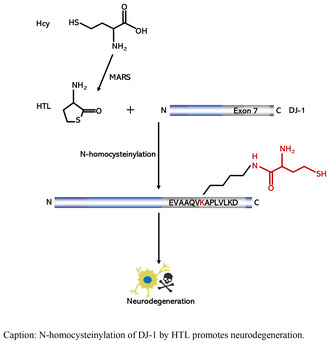

**Abbreviations**
AAVs: adeno‐associated viruses
AD: Alzheimer's disease
CCK8: cell counting kit‐8
Cyt c: cytochrome c
FBS: fetal bovine serum
HA: hemagglutinin
Hcy: homocysteine
HTL: homocysteine thiolactone
IHC: immunohistochemistry
IP: immunoprecipitation
K182: lysine 182
MAPs: microtubule‐associated proteins
MARS: methionine‐tRNA synthetase
Met: methionine
MPP^+^
: 1‐methyl‐4‐phenylpyridinium
MPTP: 1‐methyl‐4‐phenyl‐1,2,3,6‐tetrahydropyridine
MS: mass spectrometry
N‐hcy: N‐homocysteinylation
OCR: oxygen consumption rates
PD: Parkinson's disease
PEI: polyethyleneimine
PI: propidium iodide
PLA: proximity ligation assay
ROS: reactive oxygen species
SNpc: substantia nigra pars compacta
TgA53T: mice; α‐Syn A53T transgenic mice
TH: tyrosine hydroxylase
WT: wild‐type
α‐syn: α‐synuclein

## 1 INTRODUCTION

Parkinson's disease (PD) is the second most common neurodegenerative disease after Alzheimer's disease (AD) (Cacabelos, 2017). It is characterized by the loss of dopaminergic neurons in the substantia nigra pars compacta (SNpc). Oxidative stress and mitochondrial dysfunction are thought to be major causes of neurodegeneration in PD (Nagatsu et al., 2022). Converging lines of evidence suggest that DJ‐1 dysfunction is implicated in the pathogenesis of PD (Bonifati et al., 2003). DJ‐1 has multiple functions, including antioxidative stress, transcriptional regulation, chaperone activity, protease activity, and mitochondrial regulation (Ariga et al., 2013). DJ‐1 can also activate dopamine synthesis and regulate dopamine metabolism and homeostasis (Saito, 2014). Loss‐of‐function mutation of the DJ‐1 gene (Park7) causes autosomal recessive forms of PD. Thus, DJ‐1 may act as a protective factor against oxidative stress‐induced cell injury. Cells with high DJ‐1 levels are resistant to oxidative stress and PD‐related neurotoxins, while dysfunction of DJ‐1 exacerbates oxidative stress in PD (Inden et al., 2011; Liu et al., 2023).

High levels of homocysteine (Hcy) have been identified as a risk factor for PD (Bakeberg et al., 2019; Licking et al., 2017). Hcy levels are positively associated with age, disease duration, and disease severity in PD patients (Licking et al., 2017). Several potential mechanisms, such as inflammation, microvascular damage, and autoimmune responses, have been proposed to explain the biological links between hyperhomocysteinemia and PD (Lazzerini et al., 2007; Muzurović et al., 2021; Tawfik et al., 2021). Hcy exacerbates 1‐methyl‐4‐phenyl‐1,2,3,6‐tetrahydropyridine (MPTP)‐induced dopamine depletion, neuronal degeneration, and motor dysfunction (Duan et al., 2002). Furthermore, Hcy sensitizes human dopaminergic neurons to damage caused by rotenone and iron (Duan et al., 2002). However, the molecular mechanisms underlying the neurotoxicity of Hcy in PD pathogenesis have yet to be elucidated.

Homocysteine thiolactone (HTL) is an active thioester of Hcy that is catalyzed by methionyl‐tRNA synthetase (MARS) (Jakubowski, 2011). HTL has been reported to form isopeptide bonds with lysine residues in substrate proteins, a post‐translational modification known as N‐homocysteinylation (N‐hcy) (Jakubowski, 2007; Sharma et al., 2015). Previous studies have shown that in animal models of hyperhomocysteinemia, N‐hcy is correlated with increased Hcy concentrations and aging (Bossenmeyer‐Pourié et al., 2019; Zhang et al., 2018). This reaction alters protein structure and function, causes protein damage via a thiyl radical mechanism, and leads to pathological consequences (Sikora et al., 2010). For example, the N‐hcy of tau and microtubule‐associated proteins (MAPs) promotes their dissociation from β‐tubulin, affecting synaptic plasticity and cognitive function. Furthermore, protein N‐hcy in early life persists during aging (Bossenmeyer‐Pourié et al., 2019). Likewise, the N‐hcy of α‐syn exacerbates α‐syn aggregation, neurotoxicity, and dopaminergic neuronal degeneration (Zhou et al., 2022).

DJ‐1 has been identified as a substrate of N‐homocysteylation (Mei et al., 2020). In this work, we showed that the lysine 182 (K182) residue of DJ‐1 undergoes N‐hcy, which attenuates its activity against oxidative stress and mitochondrial dysfunction in 1‐methyl‐4‐phenylpyridinium (MPP^+^)‐induced SH‐SY5Y cells and MPTP‐induced PD mice. Blocking the N‐hcy of DJ‐1 at K182 restores its neuroprotective function in vitro and in vivo. Thus, our results support that N‐hcy induces DJ‐1 dysfunction and promotes neurodegeneration in PD.

## 2 RESULTS

### 2.1 HTL covalently modifies DJ‐1

**FIGURE 1:**
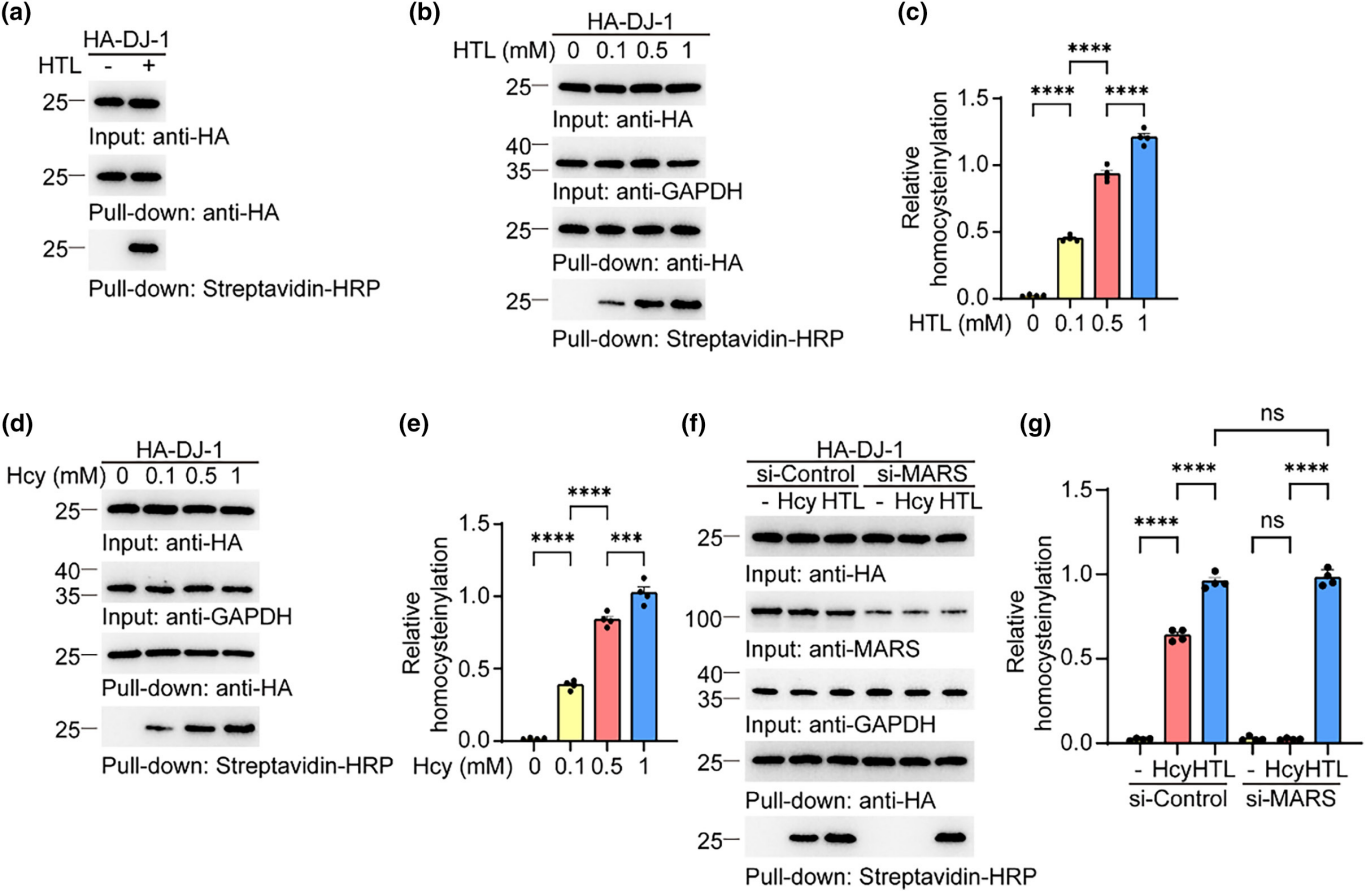
DJ‐1 is N‐homocysteinylated. HEK293 cells were transfected with HA‐DJ‐1 and incubated with vehicle, homocysteine (Hcy), or homocysteine thiolactone (HTL). (a) Chemoselective labeling of HA‐DJ‐1‐transfected HEK293 cells treated with HTL (0.1 mM) or vehicle. (b) Chemoselective labeling of HA‐DJ‐1‐transfected HEK293 cells treated with different concentrations of HTL. (c) Quantification of homocysteinylation. Results were normalized to HA. (d) Chemoselective labeling of HA‐DJ‐1 extracted from cells treated with different concentrations of Hcy. (e) Quantification of homocysteinylation. Results were normalized to HA. (f) Chemoselective labeling of HA‐DJ‐1 in cells with methionine‐tRNA synthetase (MARS) knockdown. (g) Quantification of homocysteinylation. Results were normalized to HA. Data are shown as mean ± SEM. Input: total cell lysates. Pull‐down: HA‐DJ‐1 purified from cell lysates using HA beads and labeled by chemoselective reactions. The different colored bars represent different group. *n* = 4 independent experiments. *p* values were determined by one‐way ANOVA followed by Tukey's multiple comparisons. ***p < 0.001, ****p < 0.0001, ns, not significant.

Protein N‐hcy can be labeled with azide probes (Chen et al., 2019; Zhou et al., 2022). To investigate whether DJ‐1 undergoes N‐hcy, we transfected HEK293 cells with plasmids encoding hemagglutinin (HA)‐tagged DJ‐1, and then exposed the cells to 0.1 mM HTL for 24 h. The cell lysates were subjected to chemical reaction with a biotin‐azide probe. Western blot analysis also revealed that DJ‐1 was N‐homocysteinylated in the presence of HTL (Figure 1a). Furthermore, the N‐hcy of DJ‐1 increased in an HTL concentration‐dependent manner (Figure 1b,c). Since HTL is a reactive intermediate of Hcy catalyzed by MARS, we further tested the N‐hcy of DJ‐1 induced by Hcy. Similar to HTL, exposure to Hcy also induced DJ‐1 N‐hcy in a concentration‐dependent manner (Figure 1d,e). Interestingly, knockdown of MARS eliminated Hcy‐induced N‐hcy in DJ‐1 but not HTL‐induced N‐hcy, suggesting that the conversion of Hcy to HTL is required for the N‐hcy in DJ‐1 (Figure 1f,g). Thus, our results indicate that DJ‐1 is homocysteinylated by HTL.

### 2.2 Lysine 182 is the major residue of DJ‐1 N‐hcy

**FIGURE 2:**
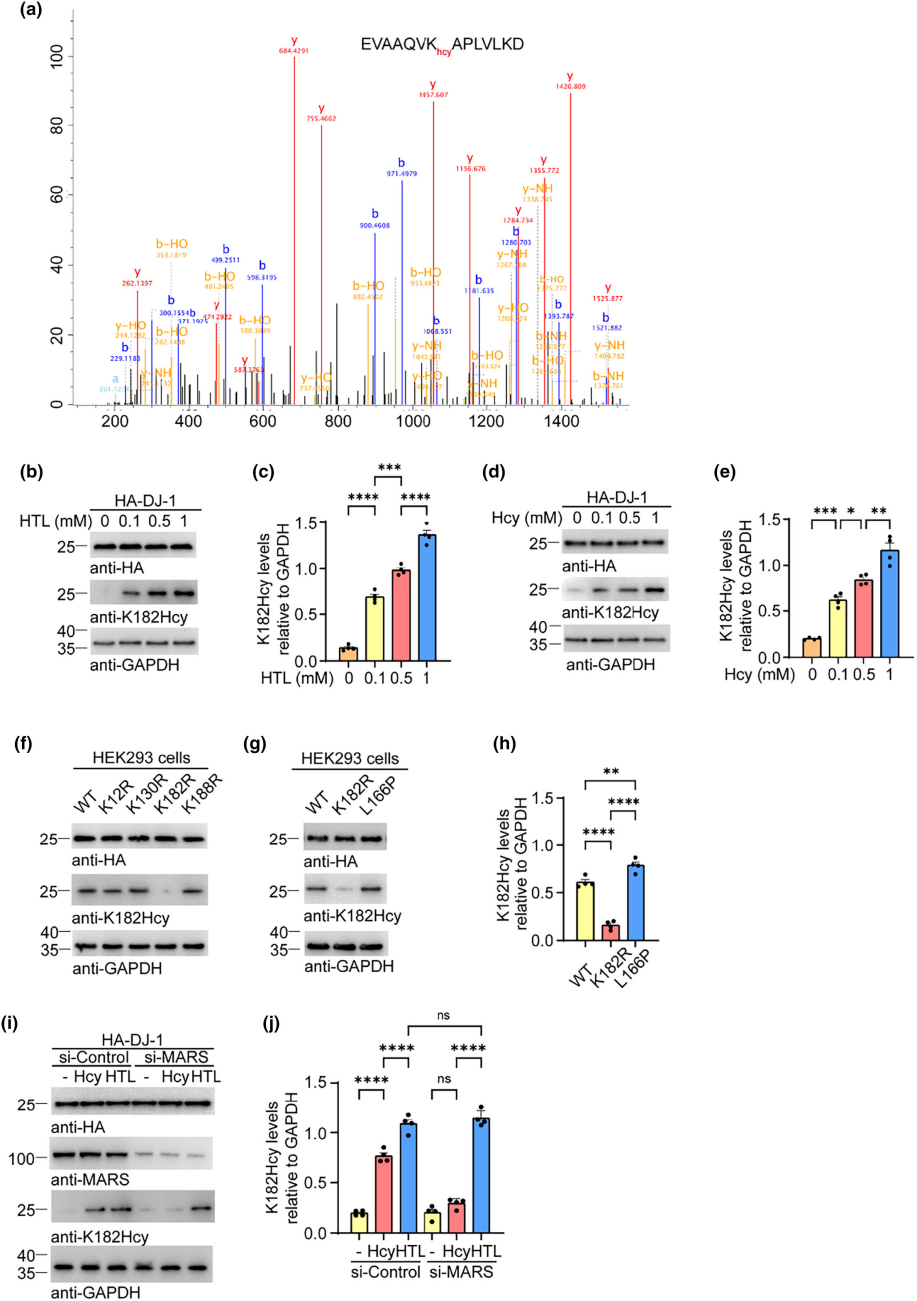
K182 is the major homocysteinylation site on DJ‐1. HEK293 cells were transfected with HA‐DJ‐1 and incubated with vehicle, homocysteine (Hcy), or homocysteine thiolactone (HTL). (a) A representative spectrum of LC‐MS/MS fragmentation containing K182 homocysteinylation. (b) Western blots showing the levels of DJ‐1 K182Hcy in cells treated with different concentrations of HTL. (c) Quantification of DJ‐1 K182Hcy. Results were normalized to GAPDH. (d) Western blots showing the levels of DJ‐1 K182Hcy in cells treated with different concentrations of Hcy. (e) Quantification of DJ‐1 K182Hcy. Results were normalized to GAPDH. (f) Western blot showing the homocysteinylation of DJ‐1 carrying K12R, K130R, K182R, and K188R mutations. (g) Western blot showing the homocysteinylation of WT DJ‐1 and DJ‐1 carrying K182R and L166P mutations. (h) Quantification of DJ‐1 K182Hcy. Results were normalized to GAPDH. (i) Western blot analysis of DJ‐1 K182Hcy in cells with methionine‐tRNA synthetase (MARS) knockdown. (j) Quantification of DJ‐1 K182Hcy. Results were normalized to GAPDH. Data are shown as mean ± SEM. *n* = 4 (b–e, g–j) independent experiments. *p* values were determined by one‐way ANOVA followed by Tukey's multiple comparisons. *p < 0.05, **p < 0.01, ***p < 0.001, ****p < 0.0001, ns, not significant.

To identify which residues of DJ‐1 are modified by N‐hcy, we treated HEK293 cells expressing HA‐tagged DJ‐1 with HTL as described above. DJ‐1 was purified using HA beads and subjected to mass spectrometry (MS). We identified homocysteinylated DJ‐1 in the immunoprecipitated samples, and multiple homocysteine lysine residues were found throughout the sequence, with K182 being the major modified residue (Figure 2a and Table S1). To study the N‐hcy of DJ‐1 at K182, we immunized rabbits with a synthetic DJ‐1 polypeptide containing a homocysteinylated lysine at K182 to produce a polyclonal antibody that recognizes homocysteinylated DJ‐1 (anti‐K182Hcy). To verify the specificity of the antibody, HEK293 cells expressing HA‐tagged DJ‐1 were exposed to HTL, immunoprecipitated with this anti‐K182Hcy antibody, and subjected to MS analysis. N‐homocysteinylated DJ‐1 at K82 was identified, supporting the specificity of this antibody (Table S2). Western blot analysis with the anti‐K182Hcy antibody detected N‐hcy of the K182 residue induced by HTL or Hcy (Figure 2b–e). Furthermore, we generated point mutant proteins that replaced lysine with arginine (K12R, K130R, K182R, K188R) and the PD‐associated L116P mutation. We found that the K182R mutation abolished DJ‐1 N‐hcy, as detected by biotin‐azide probe and the anti‐K182Hcy antibody, further confirming that K182 is the major N‐hcy residue (Figure 2f). L166P mutation of DJ‐1 induces familial PD. Interestingly, the L166P mutation enhanced the N‐hcy of DJ‐1 (Figure 2g,h). Consistent with the results obtained using the biotin‐azide probe, knockdown of MARS abrogated the K182 modification induced by Hcy but not that induced by HTL (Figure 2i,j). Taken together, these results indicate that K182 is the major N‐hcy site in DJ‐1.

### 2.3 DJ‐1 K182Hcy is increased in the PD brain in an age‐dependent manner

**FIGURE 3:**
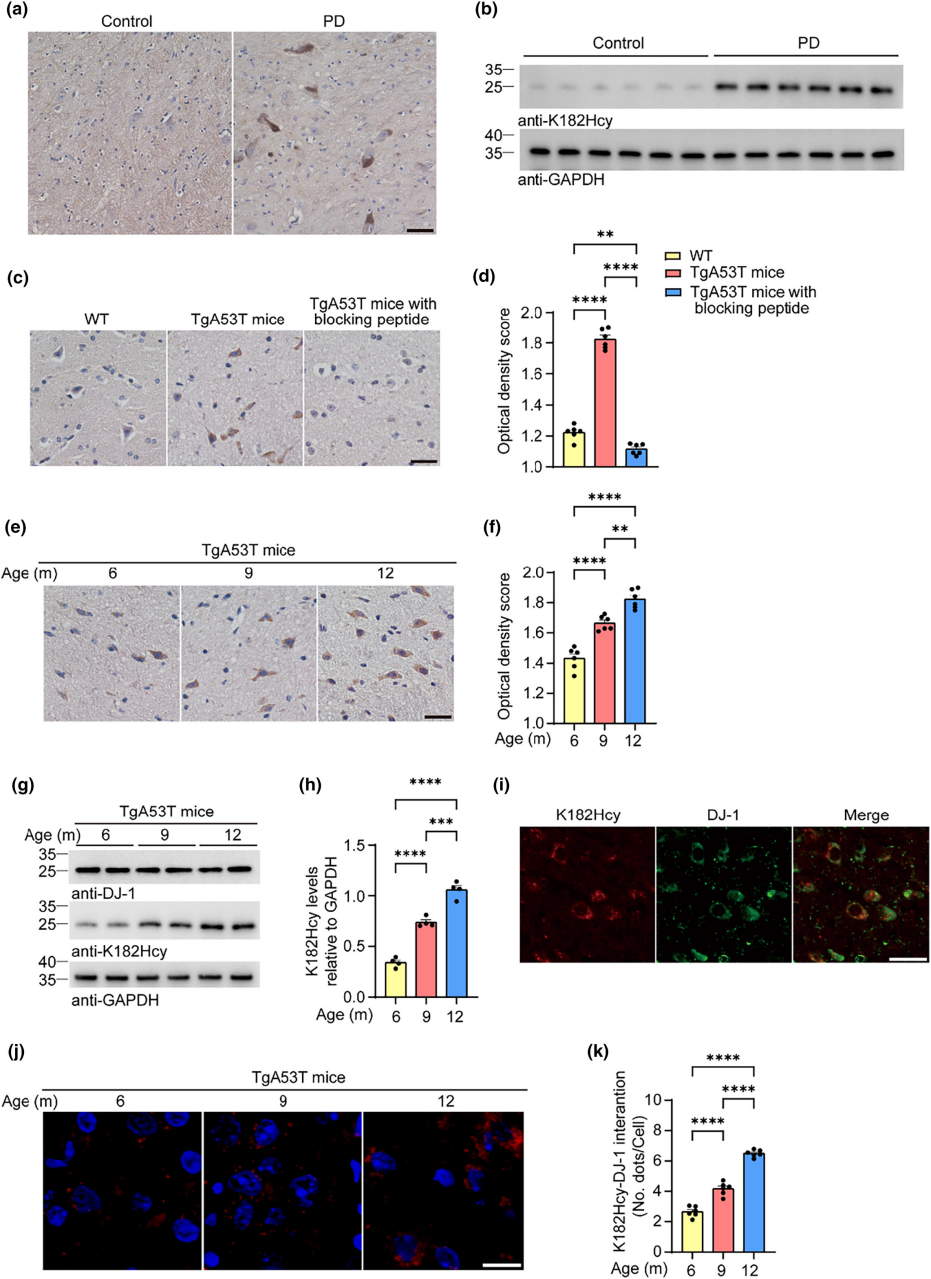
DJ‐1 K182Hcy is increased in the brain in an age‐dependent manner. (a) Immunohistochemistry of DJ‐1 K182Hcy in the SN from PD patient. (b) Western blot of DJ‐1 K182Hcy in the SN tissues from PD and control subjects. (c) Immunohistochemistry of DJ‐1 K182Hcy in the SN sections from 12‐month‐old wild‐type (WT) and TgA53T mice. (d) Quantitation of the average optical density of DJ‐1 K182Hcy in SN sections. (e) Immunostaining of DJ‐1 K182Hcy in the SN of TgA53T mice at different ages. (f) Quantitation of the average optical density of DJ‐1 K182Hcy in SN sections. (g) Western blots showing the levels of DJ‐1 K182Hcy in the SN of TgA53T mice at different ages. (h) Quantification of DJ‐1 K182Hcy. Results are normalized to GAPDH. (i) Double immunofluorescence of DJ‐1 K182Hcy (red) and DJ‐1 (green) in the SN from 12‐month‐old TgA53T mice. (j) Protein interactions between DJ‐1 and K182Hcy detected with Duolink PLA labeled in red. (k) The mean number of spots per cell as an indicator of the degree of interaction. Data are shown as mean ± SEM. *n* = 6 (c–f, j, k) or 4 (g, h) mice per group. *p* values were determined by one‐way ANOVA followed by Tukey's multiple comparisons. **p < 0.01, ***p < 0.001, ****p < 0.0001. Scale bar = 20 μm.

To detect the presence of DJ‐1 K182Hcy in the brain, we performed immunohistochemistry (IHC) using the anti‐K182Hcy antibody. Remarkably, IHC staining of the SN from PD patients and immunoblotting revealed that DJ‐1 K182Hcy was elevated in the SN from PD brains (Figure 3a,b). DJ‐1 K182Hcy was also detected in the brains of α‐syn A53T (TgA53T) transgenic mice, and the signal was blocked by pre‐incubation with the K182Hcy peptide (Figure 3c,d). Aging is the most important risk factor for PD. Interestingly, we found that the levels of both Hcy and HTL in the mouse brain increased in an age‐related manner (Zhou et al., 2022). Consistently, IHC and Western blot analyses revealed that the level of DJ‐1 K182Hcy increased during aging in the brains of TgA53T mice (Figure 3e–h). The anti‐K182Hcy signals co‐localize with DJ‐1 in brain slides (Figure 3i). The accumulation of DJ‐1 K182Hcy during aging was further confirmed using highly sensitive Duolink in situ proximity ligation assay (PLA) (Figure 3j,k).

### 2.4 N‐Hcy of DJ‐1 abolishes its antioxidant activity

**FIGURE 4:**
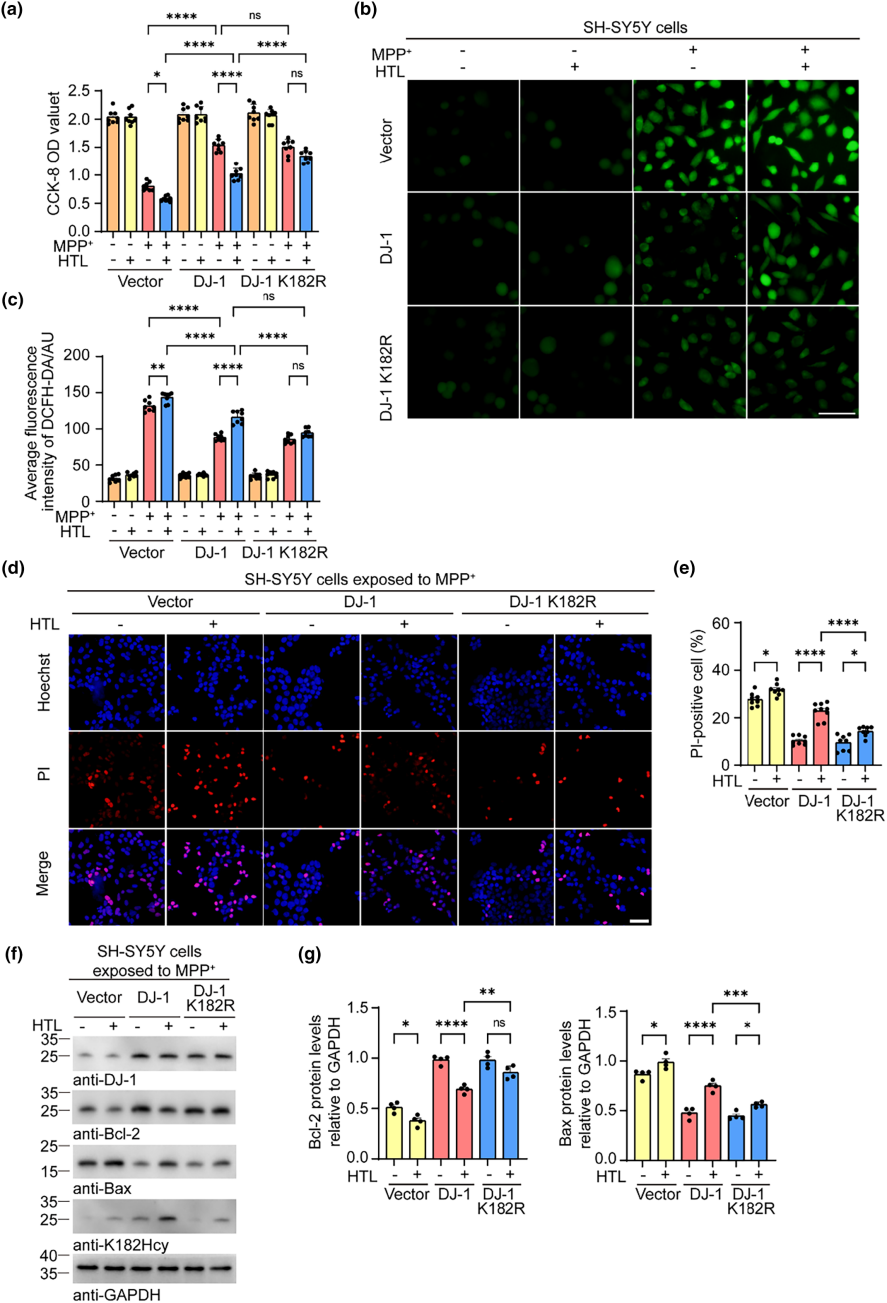
Homocysteinylation of DJ‐1 attenuates its antioxidant activity. SH‐SY5Y cells were infected with lentiviruses encoding DJ‐1 or DJ‐1 K182R, and then incubated with vehicle or 0.1 mM homocysteine thiolactone (HTL) for 24 h. (a) CCK8 analysis showing the viability of SH‐SY5Y cells expressing DJ‐1 and DJ‐1 K182R. (b) Reactive oxygen species (ROS) in SH‐SY5Y cells detected by DCFH‐DA probe after exposure to HTL and 1‐methyl‐4‐phenylpyridinium (MPP^+^). (c) Quantification of the DCFH‐DA fluorescence intensity. (d) Representative images of Hoechst and propidium iodide (PI) staining of SH‐SY5Y cells. (e) Quantification of cell death. (f) Western blots showing the levels of Bcl2 and Bax in MPP^+^‐treated SH‐SY5Y cells. (g) Quantification of Bcl2 and Bax. Results were normalized to GAPDH. Data are shown as mean ± SEM. *n* = 8 (a–e) or 4 (f, g) independent experiments. *p* values were determined by one‐way ANOVA followed by Tukey's multiple comparisons. *p < 0.05, **p < 0.01, ***p < 0.001, ****p < 0.0001, ns, not significant. Scale bar = 50 μm.

To investigate the effect of N‐hcy on the antioxidant activity of DJ‐1, we infected SH‐SY5Y cells with lentiviruses encoding wild‐type DJ‐1 and DJ‐1 K182R and treated the cells with 0.1 mM HTL for 24 h. Cell Counting Kit‐8 (CCK8) analysis showed that MPP^+^ treatment dramatically decreased cell viability and that exposure to HTL further exacerbated the neurotoxic effect of MPP^+^. Overexpression of wild‐type DJ‐1 or DJ‐1 K182R attenuated the toxic effect of MPP^+^. Interestingly, the protective effect of wild‐type DJ‐1 but not DJ‐1 K182R was abolished by treatment with HTL (Figure 4a). The levels of reactive oxygen species (ROS) and apoptosis were increased in MPP^+^‐treated cells. Overexpression of wild‐type or K182 mutant DJ‐1 partially attenuated the increase in superoxide anion levels and cell apoptosis. HTL dramatically reduced the protective effect of wild‐type DJ‐1 but not DJ‐1 K182R (Figure 4b–e). Western blot analysis of the pro‐apoptotic protein Bax and the anti‐apoptotic protein Bcl2 confirmed that HTL abolishes the protective effect of wild‐type DJ‐1 (Figure 4f,g). These results indicate that the N‐hcy of DJ‐1 at K182 abolishes its antioxidant activity and exacerbates cell injury induced by MPP^+^.

### 2.5 N‐hcy of DJ‐1 aggravates mitochondrial dysfunction

**FIGURE 5:**
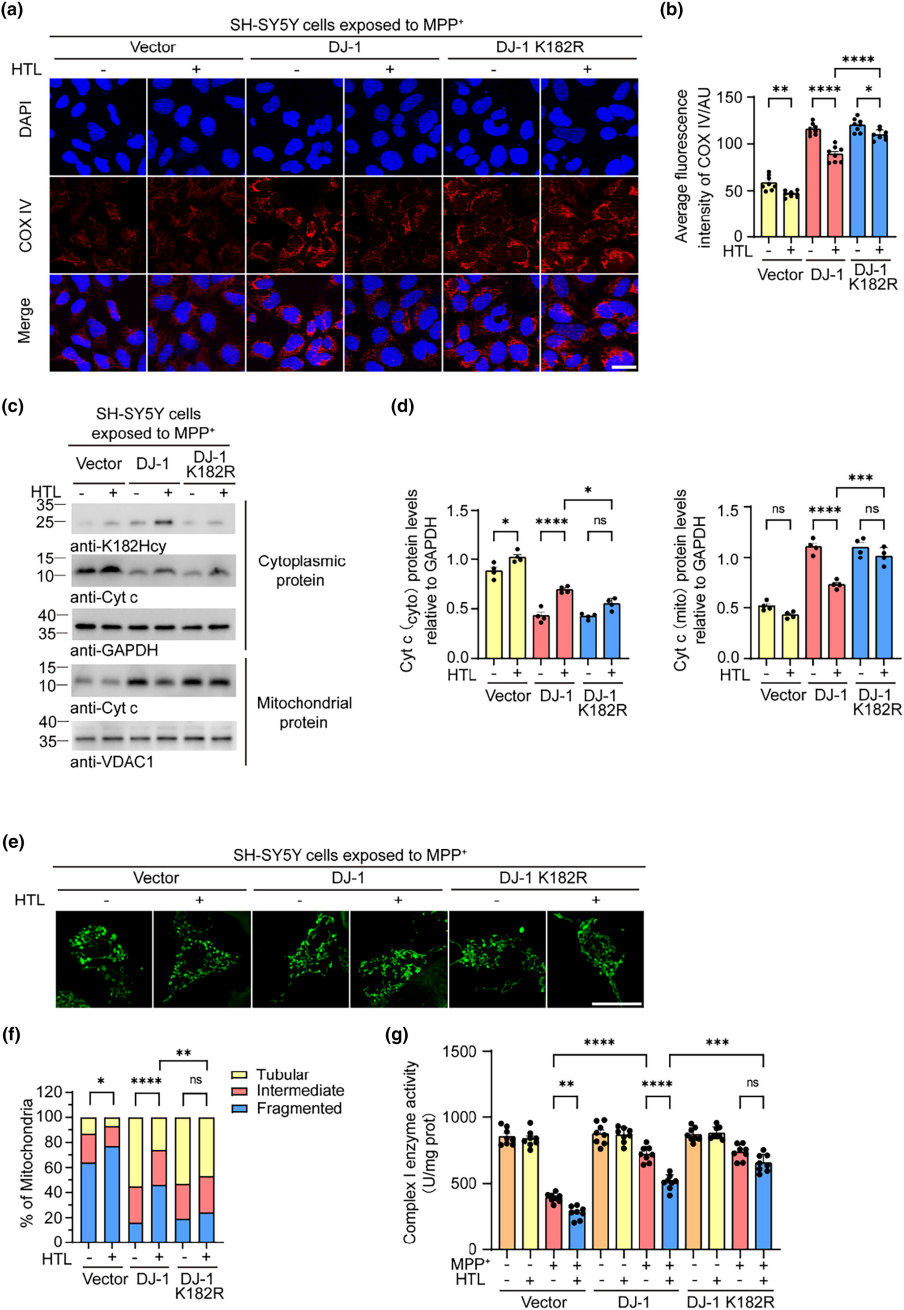
Homocysteinylation of DJ‐1 attenuates its protective effect against MPP^+^‐induced mitochondrial dysfunction. SH‐SY5Y cells were infected with lentiviruses encoding DJ‐1 or DJ‐1 K182R, and then incubated with vehicle or 0.1 mM homocysteine thiolactone (HTL) for 24 h. (a) Representative confocal microscopy images show immunostaining of Cox IV in 1‐methyl‐4‐phenylpyridinium (MPP^+^)‐treated SH‐SY5Y cells. (b) Quantitation of Cox IV fluorescence intensity. (c) Western blots showing the levels of cytochrome c (cyt c) in cytoplasmic and mitochondrial fractions. (d) Quantification of cyt c. Results are normalized to GAPDH/VDAC1. (e) Representative confocal microscopy images showing the morphology of mitochondria in MPP^+^‐induced SH‐SY5Y cells. (f) Quantification of mitochondrial morphology. The percentage of different mitochondria was quantified. (g) Complex I enzyme activity of mitochondria from SH‐SY5Y cells. Data are shown as mean ± SEM. *n* = 8 (a, b, g) or 4 (c–f) independent experiments. *p* values were determined by one‐way ANOVA followed by Tukey's multiple comparisons (a–d, g) and Kruskal–Wallis test followed by Dunn's multiple comparisons (e, f). *p < 0.05, **p < 0.01, ***p < 0.001, ****p < 0.0001, ns, not significant. Scale bar = 25 μm.

Mitochondrial dysfunction contributes to neurodegeneration in PD (Pan et al., 2022). SH‐SY5Y cells were infected with lentiviruses encoding wild‐type DJ‐1 or DJ‐1 K182R, and then exposed to 0.1 mM HTL for 24 h. Immunofluorescence staining showed that the levels of the mitochondrial marker Cox IV were lower in SH‐SY5Y cells exposed to MPP^+^. Overexpression of wild‐type DJ‐1 or DJ‐1 K182R increased the levels of Cox IV. HTL abolished the protective effect of wild‐type DJ‐1 but not that of DJ‐1 K182R (Figure 5a,b). Western blot analysis of cytochrome c (cyt c) confirmed that the release of cyt c from mitochondria was attenuated by DJ‐1 and promoted by HTL (Figure 5c,d). Overexpression of wild‐type or K182R DJ‐1 reduced the percentage of fragmented mitochondria and increased the percentage of tubular mitochondria, suggesting that DJ‐1 has a protective effect against MPP^+^‐induced mitochondrial toxicity. Again, the protective effect of wild‐type DJ‐1, but not K182R DJ‐1, on mitochondria was abolished by HTL (Figure 5e,f). Mitochondrial complex enzymatic activity assays showed that overexpression of wild‐type DJ‐1 or DJ‐1 K182R attenuated the dysfunction of mitochondrial complexes I and IV induced by MPP+, but not complexes II, III, and V. Interestingly, HTL abolished the protective effect of wild‐type DJ‐1 but not DJ‐1 K182R (Figure 5g and Figure S1a–d). Similar results were observed by oxygen consumption rate (OCR) assay. The basal respiration and maximal respiration were impaired by MPP+. HTL abolished the protective effect of wild‐type DJ‐1 but not K182R mutant DJ‐1 (Figure S2a,b). These results suggest that N‐hcy of DJ‐1 at K182 partially abolishes its protective effect against mitochondrial dysfunction induced by MPP^+^.

### 2.6 L‐methionine administration exacerbates PD pathology induced by MPTP

**FIGURE 6:**
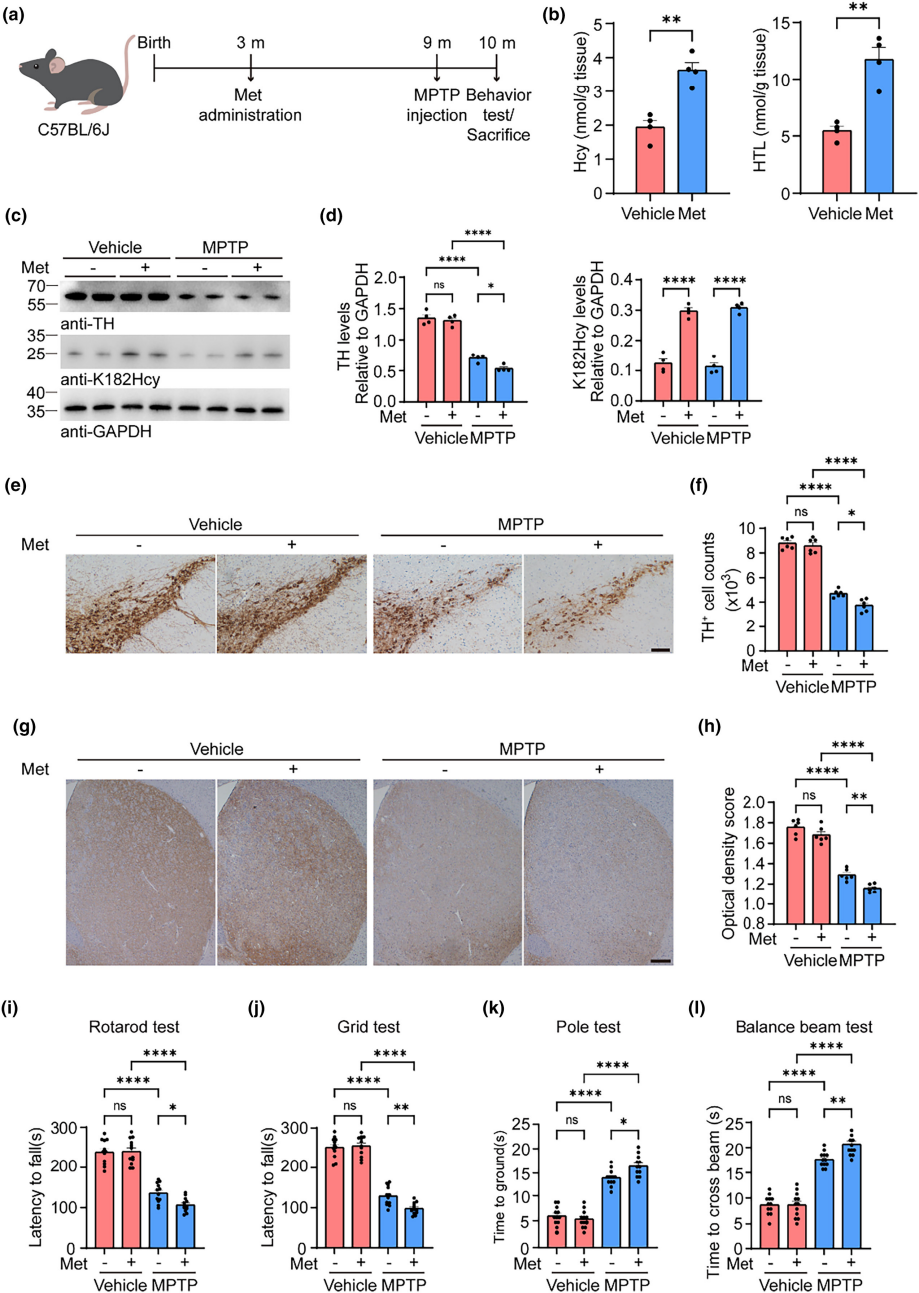
L‐Methionine administration exacerbates MPTP‐induced neurodegeneration and motor impairments. The mice were treated with 0.5% L‐methionine (Met) for 6 months and then injected with 1‐methyl‐4‐phenyl‐1,2,3,6‐tetrahydropyridine (MPTP). (a) Animal experiment process and timeline chart. (b) Brain tissues were assayed by LC‐MS for levels of Hcy and GC‐MS for levels of HTL. (c) Levels of tyrosine hydroxylase (TH) and DJ‐1 K182Hcy in the striatum (Str) of wild‐type (WT) mice. (d) Quantification of TH and DJ‐1 K182Hcy. Results are normalized to GAPDH. (e) TH immunohistochemistry images in the substantia nigra pars compacta (SNpc). Scale bar = 100 μm. (f) Counting of TH‐positive cells in the SNpc. (g) TH immunohistochemistry images in the striatum. Scale bar = 200 μm. (h) Quantitation of average optical densities of dopaminergic terminals in the striatum. (i–l) Behavioral tests. Shown are the results of rotarod test (i), grid test (j), pole test (k), and balance beam test (l). Data are shown as mean ± SEM. *n* = 4 (b–d), 6 (e–h), 12 (i–l) mice per group. *p*‐values were determined by one‐way ANOVA followed by Turkey's multiple comparisons. *p < 0.05, **p < 0.01, ****p < 0.0001, ns, not significant.

Hcy is a byproduct of the methionine metabolism pathway. Administration of L‐methionine (Met) increases the levels of Hcy and HTL in the mouse brain (Zhou et al., 2022). Thus, we further tested the effect of Met administration on mice injected with MPTP, one of the most widely used neurotoxins for inducing PD‐like dopaminergic neurodegeneration in mice. The mice were treated with Met for 6 months and then injected with MPTP (30 mg/kg) for five consecutive days. The mice were sacrificed 3 weeks after MPTP injection (Figure 6a). As expected, Met administration increased the levels of Hcy, HTL, and DJ‐1 K182Hcy in the mouse brain (Figure 6b–d). The number of tyrosine kinase (TH)‐positive neurons in the SN and the density of TH‐positive fibrils in the stritum were decreased in mice injected with MPTP, indicating degeneration of the nigrostriatal dopaminergic pathway. Interestingly, Met administration exacerbated neurodegeneration in mice injected with MPTP (Figure 6e–h). Behavioral tests, including the rotarod test, grid test, pole test, and balance beam test, revealed that mice injected with MPTP had impaired motor function compared with vehicle‐treated mice. PD‐like motor impairment was exacerbated by Met treatment (Figure 6i–l). Taken together, these findings suggest that the administration of Met exacerbates MPTP‐induced neurodegeneration and motor impairments in mice.

### 2.7 Blocking the N‐hcy of DJ‐1 ameliorates the toxic effect of hyperhomocysteinemia

**FIGURE 7:**
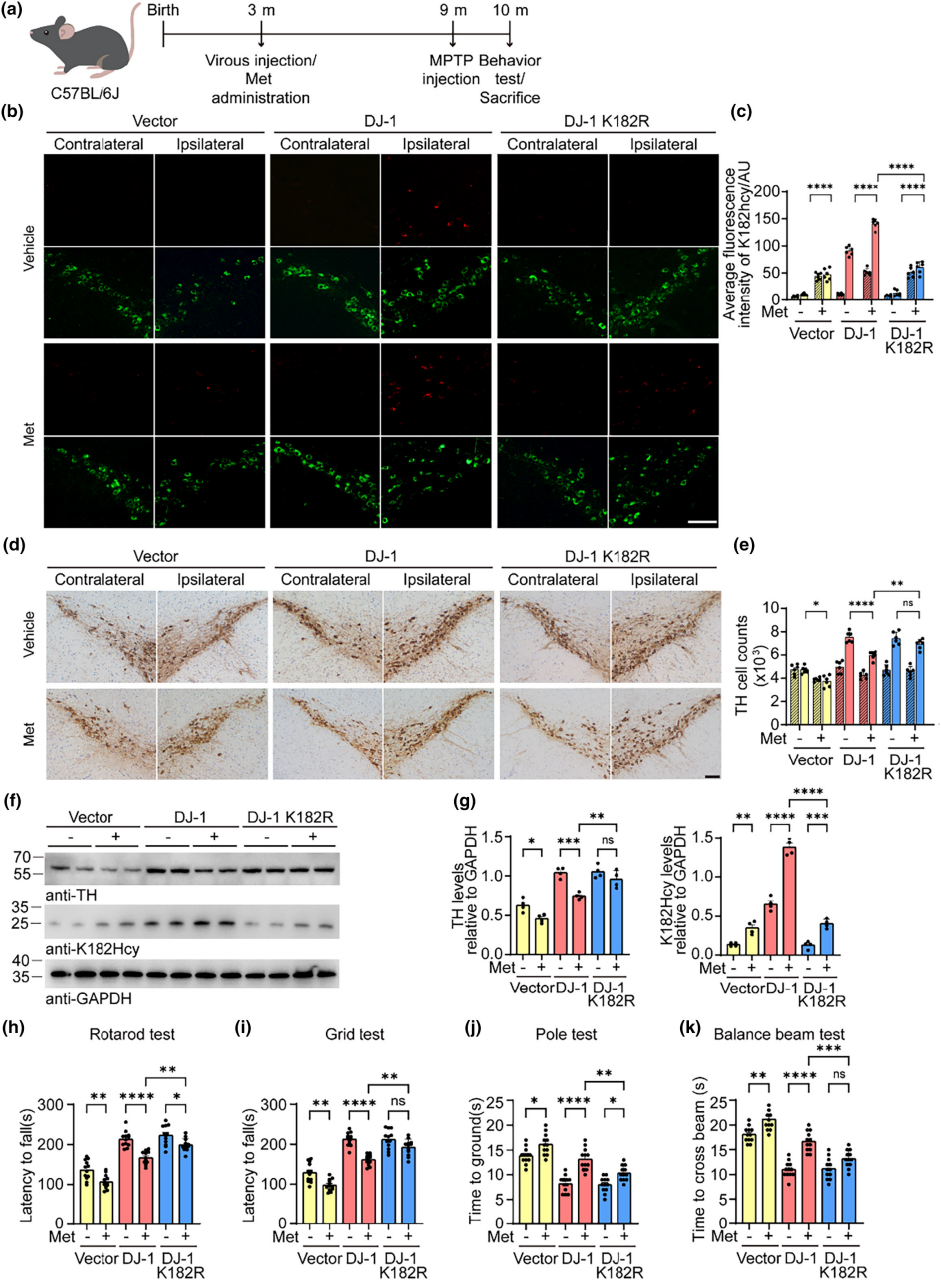
Blocking the N‐homocysteinylation of DJ‐1 ameliorates the toxic effect of hyperhomocysteinemia. AAV‐DJ‐1, AAV‐DJ‐1 K182R, or control AAVs were injected into the substantia nigra (SN) of wild‐type mice. The mice were treated with L‐methionine (Met) for 6 months and then injected with MPTP (30 mg/kg/d for 5 days). (a) Animal experiment process and timeline. (b) Representative double immunostaining for DJ‐1 K182Hcy (red) and tyrosine hydroxylase (TH) (green) in the substantia nigra pars compacta (SNpc). (c) Quantitation of DJ‐1 K182Hcy fluorescence intensity in the contralateral (filled) and ipsilateral (blank) SNpc. (d) Representative TH immunohistochemistry images of the SNpc. (e) Stereological counting of the number of TH‐positive neurons in the contralateral (filled) and ipsilateral (blank) SNpc. (f) Western blots showing the levels of TH and DJ‐1 K182Hcy in the ipsilateral ventral midbrain. (g) Quantification of TH and DJ‐1 K182Hcy. Results are normalized to GAPDH. (h–k) Behavioral teats. Shown are the results of the rotarod test (h), grid test (i), pole test (j), and balance beam test (k). Data are shown as mean ± SEM. *n* = 6 (b–e), 4 (f, g), or 12 (h–k) mice per group. *p* values were determined by one‐way ANOVA followed by Tukey's multiple comparisons. *p < 0.05, **p < 0.01, ***p < 0.001, ****p < 0.0001, NS, not significant. Scale bar = 100 μm.

To determine whether the DJ‐1 K182Hcy modification mediates the toxic effect of Hcy in vivo, we injected adeno‐associated viruses (AAVs) encoding DJ‐1 or DJ‐1 K182R into the right SN of three‐month‐old mice and fed the mice Met in the drinking water for 6 months. The mice were then injected with MPTP for five consecutive days (Figure 7a). Three weeks after MPTP injection, the expression of exogenous DJ‐1 in the SN was confirmed by immunostaining with an anti‐DJ‐1 antibody (Figure S3a). Western blot analysis also revealed that the levels of DJ‐1 and DJ‐1 K182R in the brain were comparable (Figure S3b,c). Met treatment increased the level of DJ‐1 K182Hcy. Double immunostaining and IHC showed that MPTP induced the loss of TH‐positive neurons in the SN and striatum (Figure 7b–e and Figure S4a,b). Overexpression of DJ‐1 and DJ‐1 K182R attenuated the loss of TH‐positive neurons induced by MPTP. Met treatment partially abolished the protective effect of DJ‐1 but not DJ‐1 K182R. These results were further confirmed by Western blot analysis (Figure 7f,g). According to the results of behavioral analysis, including the grid test, rotarod test, balance test, and pole test, overexpression of DJ‐1 and DJ‐1 K182R alleviated the PD‐like motor impairment induced by MPTP. Met exacerbated behavioral deficits in mice expressing DJ‐1 but not in mice expressing DJ‐1 K182R (Figure 7h–k). These results suggest that overexpression of the K182R mutation in DJ‐1 partially alleviates the toxic effect of Hcy compared to that in wild‐type DJ‐1.

## 3 DISCUSSION

In the present study, we demonstrated that N‐hcy of DJ‐1 at K182 results in the loss of its protective effect. The levels of DJ‐1 K182Hcy increase in an age‐dependent manner, consistent with the increased levels of Hcy and HTL in the brain during aging. DJ‐1 K182Hcy interferes with the activity of DJ‐1 to attenuate oxidative stress and mitochondrial dysfunction. In the MPTP mouse model of PD, chronic exposure to Met leads to decreased ability of DJ‐1 to protect dopaminergic neurons in the SN. Blockade of DJ‐1 N‐hcy by mutating the K182 residue partially preserved the protective effect of DJ‐1. The incomplete functional recovery of K182R DJ‐1 indicates that factors other than DJ‐1 homocysteinylation may also contribute to the toxic effect of Met. Our findings are in agreement with epidemiological evidence that hyperhomocysteinemia is a risk factor for PD (Licking et al., 2017; Saadat et al., 2018; Sapkota et al., 2014). Together, this evidence supports that the N‐hcy of DJ‐1 is involved in neurodegeneration in PD patients.

It has been reported that high levels of Hcy are closely related to the development and progression of PD (Martignoni et al., 2007). Elevated Hcy levels are associated with more severe motor impairment, depression, and cognitive dysfunction in PD (O'Suilleabhain et al., 2004). One of the pathogenic processes related to Hcy‐induced neurotoxicity might involve modifications of protein structure (Lehotský et al., 2016). Hcy is converted to HTL in an error‐editing reaction catalyzed by MARS. HTL forms isopeptide bonds with protein lysine residues known as N‐hcy (Jakubowski, 2019). Emerging lines of evidence suggest that N‐hcy plays a role in regulating the function of proteins and is associated with cytotoxic, proinflammatory, and proatherogenic effects linked to cardiovascular disease, diabetes, etc (Jakubowski et al., 2009; Tripathi et al., 2022).

Here we show that DJ‐1 can be homocysteinylated using multiple methods, including bisorthorhomide probes, LC‐MS/MS, and a novel polyclonal antibody against DJ‐1 K182Hcy. Overexpression of DJ‐1 in cells protects mitochondrial function and regulates dopamine metabolism and homeostasis (Saito, 2014; Zhang et al., 2016). Interestingly, we found that N‐hcy of DJ‐1 inhibits its protective function, while the K182R mutation that blocks N‐hcy partially attenuates the detrimental effect of HTL. Among the multiple lysine residues in DJ‐1, K182 was detected as the major residue modified by N‐homocysteine. It is conceivable that the amino acids surrounding lysine residues may affect the selectivity of N‐hcy.

N‐homocysteinylated tau and MAP1 have been identified in protein aggregates in the brains of patients with AD and vascular dementia. Strikingly, protein N‐hcy was higher in the brains of 450‐day‐old rats that were exposed to vitamin B12 and folate deficiency during gestation and lactation and returned to a normal diet at weaning (Bossenmeyer‐Pourié et al., 2019). These results suggest that cumulative protein N‐hcy may play a role in brain aging and neurodegenerative diseases. Our previous experiments showed that increases in Hcy and HTL in the brain are accompanied by the accumulation of homocysteinylated α‐syn (Zhou et al., 2022). We showed that Met administration also enhances DJ‐1 K182Hcy and exacerbates PD‐like motor impairment. This finding is consistent with the clinical observation that higher Hcy levels are associated with motor and cognitive decline in PD patients (Sleeman et al., 2019). The N‐hcy of DJ‐1 and α‐syn may synergistically contribute to PD progression.

The rate of protein N‐hcy is strongly correlated with the concentration of the protein and HTL (Chen et al., 2018; Jakubowski, 1999). Furthermore, the reactivity of HTL has also been shown to depend on the pKa of the amino acid side chains (Garel & Tawfik, 2006). Despite a large body of data, it is not yet clear what determines the extent of structural and functional alterations in proteins due to N‐hcy. It is conceivable that modifications of specific lysine residues may alter the physicochemical properties of substrate proteins (Kumar et al., 2014). Occupation of Lys residues by N‐hcy may also prevent other post‐translational modifications with regulatory functions, such as methylation, ubiquitination, or acetylation. Furthermore, DJ‐1 has chaperone and protease activities and is regulated by its carboxy‐terminal helical structure (Tao & Tong, 2003). K182 is located in helix αH near the extreme C‐terminus of DJ‐1, indicating that its homocysteinylation may affect the function of DJ‐1. Since the L166P mutation can change the structure and activity of DJ‐1 (Moore et al., 2003), we can speculate that this mutation affects the extent of homocysteinylation at the K182 site of DJ‐1. Indeed, we observed that the PD‐associated L166P mutation of DJ‐1 enhances N‐homocysteinylation, suggesting that the detrimental effect of the L166P mutation may at least in part be attributed to N‐homocysteinylation.

This study has several limitations. In the in vitro experiments, the cells were treated with 100 μM Hcy or HTL to trigger the homocysteinylation of DJ‐1. The concentrations are higher than those found in brain tissues and cerebrospinal fluid (<15 μM) (Zhang et al., 2022). Thus, caution should be used when extrapolating the results on the pathophysiology of PD. Furthermore, in SH‐SY5Y cells, the K182R mutation in DJ‐1 only partially attenuated the neurotoxicity of HTL, indicating that factors other than DJ‐1 homocysteinylation may contribute to the toxic effect of HTL. In animal models, we found that the administration of Met exacerbates PD‐like phenotypes induced by MPTP, which was partially attenuated by the K182R mutation in DJ‐1. Thus, increased concentrations of Hcy may contribute to the onset and progression of PD. It should be noted that administration of Met may also trigger other pathological changes in addition to protein N‐hcy modification.

In conclusion, we showed that N‐hcy of DJ‐1 K182 abolishes the physiological function of DJ‐1 and promotes neurodegeneration. The discovery of DJ‐1 N‐hcy in PD may provide a potential biomarker for the diagnosis of PD and a new target for the development of therapeutic interventions.

## 4 MATERIALS AND METHODS

### 4.1 Animals and MPTP treatment

Adult C57BL/6J male mice and the human α‐syn A53T transgenic line M83 were obtained from the Jackson Laboratory (stock numbers: 000664 and 004479). The mice were kept under SPF conditions with a 12‐h light/dark cycle at 22°C and were fed food ad libitum. Three‐month‐old mice were randomly assigned to each experimental group. The sample size was determined by Power and Precision (Biostat). Mice were treated intraperitoneally with MPTP (30 mg/kg) or an equivalent volume of saline daily for five consecutive days to induce the PD model at 9 months of age (Jackson‐Lewis & Przedborski, 2007). The behavioral tests were performed 21 days after administration. The experimental procedures were approved by the Institutional Animal Care and Use Committee (IACUC) of Renmin Hospital of Wuhan University, with the IACUC issue number of the WDRM animal (welfare) 20220207A.

### 4.2 Met administration in mice

The mice in the Met group were administered 0.5% L‐methionine (wt/vol, dissolved in drinking water) for 6 months beginning at 3 months of age. The mice in the control group received normal drinking water.

### 4.3 Cell culture and treatment

HEK293 cells and SH‐SY5Y cells were tested for mycoplasma contamination before use and cultured in Dulbecco's modified Eagle's medium (DMEM) containing 10% fetal bovine serum (FBS) and 100× penicillin/streptomycin at 37°C under 5% CO_2_. Hcy (Sigma, 69,453) and HTL (Sigma, H6503) were freshly prepared before use. After the cells were starved in 1% FBS medium for 24 h, Hcy or HTL was added to the culture media to reach the final indicated concentrations (0.1, 0.5, and 1 mM) as previously described (Zhou et al., 2022). To induce a PD‐like phenotype, the cells were treated with 1 mM MPP^+^ iodide (Sigma, D048) diluted in PBS for 24 h.

### 4.4 Human tissue samples

Post‐mortem brain samples from pathologically diagnosed PD patients (*n* = 6) and control individuals (*n* = 6) with no neurological conditions were obtained from the Emory Alzheimer's Disease Research Center. PD patients were clinically diagnosed and neuropathologically confirmed. Informed consent was obtained from all subjects. The average ages of the control and PD patients were 71.8 and 71.2 years, respectively. The average disease duration was 6.8 years. The study was approved by the biospecimen committee of Emory University.

### 4.5 Plasmid constructs and transfection

Cells were transfected with plasmids encoding HA‐tagged wild‐type (WT), point‐mutant DJ‐1 or control plasmids using polyethyleneimine (PEI). For MARS knockdown, HEK293 cells were transfected with siRNAs using Lipofectamine 2000 (Invitrogen, 11,668,019). The siRNA sequences used were as follows: sense: 5′‐CCGCUGGUUUAACAUUUCGUU‐3′, antisense: 5′‐ACGAAAUGUUAAACCAGCGG‐3′.

### 4.6 Lentiviral infection of SH‐SY5Y cells

To overexpress DJ‐1 and DJ‐1 K182R, lentiviruses encoding wild‐type DJ‐1 and DJ‐1 K182R under the control of the CMV promotor were produced by BrainVTA (Wuhan, China). The lentivirus titer was 2 × 10 (Licking et al., 2017) TU/mL. SH‐SY5Y cells were infected with lentivirus when reaching 70% confluence. Stable single clones were obtained after three passages. DJ‐1 expression in the stably infected cells was confirmed using western blot analysis.

### 4.7 Western blot analysis

Cells were lysed with ice‐cold NP‐40 lysis buffer containing a cocktail of protease and phosphatase inhibitors. Dissection of the SN from mice was performed as previously described (Salvatore et al., 2012). Tissues were homogenized in ice‐cold RIPA lysis buffer containing protease and phosphatase inhibitors. The lysates were then sonicated briefly and centrifuged at 15,000 r.p.m. for 20 min. The protein concentration was measured using the BCA assay. Protein extracts were separated by 10% Bis‐Tris SDS‐PAGE gels. The following primary antibodies were used: HA (1:5000; Proteintech, 51064‐2‐AP), GAPDH (1:8000; Proteintech, 60004‐1‐Ig), streptavidin‐HRP (1:5000; Proteintech, SA00001‐0), MARS (1:10000; Proteintech, 14829‐1‐AP), DJ‐1 (1:1000; Abcam, ab76008), Bax (1:1000; HUABIO, ET1603‐34), Bcl‐2 (1:1000; Abcam, ab218123), Cyt c (1:1000; Abcam, ab113504), VDAC1 (1:1000; Santa Cruz, sc‐390996) and TH (1:1000; Abcam, ab117112). The following secondary antibodies conjugated to horseradish peroxidase (HRP) were used: HRP‐conjugated anti‐mouse IgG (1:8000; Bio‐Rad, 1706516) and HRP‐conjugated anti‐rabbit IgG (1:8000; Bio‐Rad, 1706515). The membranes were blocked in 5% skim milk for 1 h at room temperature and incubated with primary antibodies overnight at 4°C. Then, the membranes were washed 5 times in TBST and incubated with HRP‐conjugated secondary antibodies for 1 h at room temperature. Signals were developed by detecting enhanced chemiluminescence (ECL) with an imaging system (Bio‐Rad, ChemiDoc™ Touch). The band intensity was analyzed and quantified using ImageJ.

### 4.8 Immunoprecipitation

The samples were lysed in PBS buffer containing 1% Triton X‐100 and protease inhibitors, sonicated briefly, and centrifuged at 15,000 r.p.m. for 20 min. The supernatant was incubated with an antibody or control IgG at room temperature and mixed (on a shaker or rotator) for 2 h. Protein A MagBeads (L00273, GenScript) were added to the mixture and incubated overnight at 4°C. The beads were collected using a magnetic separation rack and washed three times with PBS. After washing, the bound proteins were eluted from the beads by boiling in sample buffer and subjected to SDS–PAGE analysis.

### 4.9 Immunohistochemistry and immunofluorescence

The procedures used were the same as those we reported previously (Pan et al., 2022). Briefly, mouse brain tissues were removed, infiltrated with paraffin, and cut into serial 4 μm sections. The slices were processed using an IHC Detection System Kit (ZSGB‐BIO, PV‐6001/PV‐6002). The signal was developed using DAB. For immunofluorescence staining, the samples were stained with corresponding Alexa Fluor 594‐ or 488‐conjugated secondary antibodies (1:1000; Invitrogen). Nuclei were stained with DAPI (1 μg/mL, BioFroxx, 1155MG010) for 5 min. The sections were incubated with primary antibodies against Cox IV (1:500, Abcam, ab16056), TH (1:1000, Abcam, ab117112), or K182Hcy (1:500, Abmart) at 4°C overnight. Images were captured using an Olympus DP80 microscope equipped with TH4‐200 and U‐HGLGPS light sources. The levels of immunoreactivity were determined by optical density analysis using ImageJ plus the IHC Profiler plugin and measured using the procedure in the literature as follow: optical density score = percentage contribution of high positive × 4 + percentage contribution of positive × 3 + percentage contribution of low positive × 2 + percentage contribution of negative × 1 (Seyed Jafari & Hunger, 2017).

### 4.10 Stereological quantification of TH‐positive neurons and striatal terminals

Unbiased stereological counts of TH‐positive neurons within the SNpc were performed using stereological principles. Every sixth section from the caudal to rostral boundaries of the SN was subjected to the counting procedure. TH‐positive fiber densities in the striatum were analyzed with ImageJ plus the IHC Profiler plugin.

### 4.11 Cell death and viability assessment

The percentage of cell death was determined by staining 12‐well plates with Hoechst 33342 and propidium iodide (PI) (C1056‐1; Beyotime). After MPP^+^ treatment for 24 h, the cells were incubated with staining buffer containing Hoechst staining solution and PI staining solution in an ice bath at 4°C for 20–30 min in a 5 μL working solution/1 mL buffer ratio. The cells were washed with PBS three times and then observed under a fluorescence microscope. Cell viability was analyzed using the Cell Counting Kit‐8 viability assay kit (CK04, Solarbio). Briefly, cells were plated at a density of 1 × 10^4^ cells/100 μL in 96‐well plates and exposed to MPP^+^. After treatment, 10 μL of CCK‐8 solution was added to each well. The plates were incubated for 2 h at 37°C. The medium was removed, and the cells were washed twice with PBS. The absorbance at 450 nm was measured using a SpectraMax Plus 384 microplate reader. Cell viability was expressed as percentage versus the control group.

### 4.12 Determination of intracellular ROS levels

Determination of intracellular ROS was performed using ROS assay kit (Jiancheng Bioengineering Institute, Nanjing, China) according to the manufacturer's instructions. The cells were plated in 12‐well plates and exposed to MPP^+^ for 24 h. The culture medium was removed, and 1 mL of DCFH‐DA (10 μm) diluted in serum‐free medium at 1:1000 was added. The cells were incubated at 37°C for 30 min and then gently washed with serum‐free culture medium. Finally, the fluorescence intensity was detected by an Olympus inverted fluorescence microscope (Olympus TH4‐200, Japan) at an excitation wavelength of 488 nm. The results were expressed as fluorescence values.

### 4.13 Mitochondrial isolation and complex I–V enzyme activity assay

Mitochondria were isolated from SH‐SY5Y cells using a cell mitochondria isolation kit (SM0020, Solarbio). The cells were resuspended in precooled lysis buffer. The cell suspension was transferred to a small‐volume glass homogenizer and ground 30 times within an ice bath. The cell suspension was homogenized and centrifuged at 1000 × g for 10 min at 4°C. The resultant supernatant was then centrifuged at 12,000 × g for 10 min at 4°C. The obtained mitochondrial precipitates were resuspended in storage buffer and used immediately. Protein concentrations were determined by BCA assay (Thermo Fisher). Mitochondrial respiration complex activity was measured using the complex I–V enzyme activity microplate assay kit (BC0510, BC3230, BC3240, BC0940, and BC1440; Solarbio). The enzyme activity assays were conducted according to the manufacturer's instructions. Briefly, mitochondrial homogenates were added to the respective reaction buffer. The reaction mixture was transferred to a pre‐warmed quartz cuvette and immediately placed into a spectrophotometer. Mitochondrial complex activity was expressed as nmol/min/mg protein.

### 4.14 Duolink in situ PLA

Duolink in situ PLA was performed as previously described (Alam, 2018). Briefly, after the same processes of washing, permeabilizing, and blocking as described for histological analysis, sections were incubated with anti‐DJ‐1 (1:200) and anti‐K182Hcy (2:100) primary antibodies overnight at 4°C. PLA secondary probes were then added and incubated at 37°C for 1 h in the dark. The slices were further washed twice with PLA wash buffer A. Finally, ligation and amplification were carried out using a Duolink PLA Kit (Sigma‐Aldrich) according to the manufacturer's protocol. Duolink in situ Detection Reagent Red was used for detection. Images were captured using an Olympus inverted fluorescence microscope (Olympus TH4‐200, Japan). The mean number of spots per cell was calculated for each treatment and used as an indicator of the extent of the DJ‐1‐K182Hcy interaction.

### 4.15 Mitochondrial morphological analysis

SH‐SY5Y cells expressing DJ‐1 or DJ‐1 K182R were transfected with the Mito‐Dendra2 plasmid. Morphological analysis was conducted using a Leica TCS SP8 confocal microscope. The images were randomized, and the prevailing mitochondrial morphology in each cell was classified as tubular, fragmented, or intermediate by investigators who were blinded to the group allocation. Mitochondria were classified as tubular when their length was >1 μm, fragmented when their length was <0.5 μm, and intermediate when their length was between 0.5 and 1 μm.

### 4.16 Mitochondrial OCR measurement

Mitochondrial OCR was measured using Seahorse XF Analyzer (Agilent Technologies, Santa Clara, CA, USA) following the manufacturer's protocol as previously described (Zhang & Zhang, 2019). We inoculated the cells in a 96‐well Seahorse assay plate. The cells were incubated overnight in a 37°C incubator. The next day, the cells were washed and supplied with assay medium (supplemented with 10 mM glucose, 1 mM pyruvate, and 2 mM glutamine, adjusted to pH 7.4), and the cell plates were placed in a non‐CO2 37°C incubator for 1 h. After baseline was measured, oligomycin, FCCP, and rotenone/antimycin A solutions were sequentially added to each well. The basal OCR was calculated based on the area under the curve before the injection of oligomycin. The maximal OCR was calculated based on the area under the curve (AUC) between the injection of FCCP and rotenone/antimycin A.

### 4.17 Detection of Hcy and HTL levels in brain tissue

All the analyses were performed by Sensichip Biotech Company (Sensichip Biotech Co., Ltd.). The concentration of Hcy was performed by liquid chromatography‐massspectrometry (LC‐MS) analysis. Brain tissues were added with 20 μL of 500 mM DTT, 380 μL of extraction solution (40:40:20 acetonitrile: methanol: water). Samples were swirled, ground, incubated at room temperature for 30 min, sonicated at 4°C for 10 min, and centrifuged at 4°C for 13,523 g for 10 min. Then, the supernatants were evaporated, reconstituted with 150 μL of 60:40 acetonitrile: water, vortexed well, and clarified by centrifugation at 13,523 g for 10 min at 4°C. The supernatants were transferred to an injection vial for analysis. The concentration of HTL was performed by using Thermo Trace 1300 gas chromatography system coupled with ISQ7000 mass spectrometry (GC‐MS) as previously described (Piechocka et al., 2020).

### 4.18 Behavioral tests

The motor function of the mice was monitored by the rotarod test, pole test, and balance beam test. In the rotarod test, mice were placed on a spinning rod with a spinning rate gradually accelerated from 5 to 40 r.p.m. within 5 min. The latency to fall off was recorded. In the pole test, the mice were placed head up on the top of a vertical rough wooden pole (75 cm long, 1 cm diameter) and allowed to descend autonomously. The mice were trained for two consecutive days consisting of three test trials each day. The time from turning to reaching the base of the pole was recorded. In the grid test, the mice were placed on a horizontal wire grid. The grid was lightly shaken to make the mice grab the grid and then turned upside down. The latency of the mice to fall off was recorded. Trials were stopped if the mice remained on the grid for 5 min. The beam‐walking test was performed to assess motor coordination and balance. The mice were placed on one end of a narrow beam and were allowed to escape to the other end autonomously. The time to cross the beam (2 × 100 cm) was recorded.

### 4.19 Viral construction and stereotaxic injection

AAV particles encoding human WT and K182R mutant DJ‐1 under the control of the human synapsin I gene promoter were prepared by BrainVTA (Wuhan, China). Unilateral intracerebral injection of AAVs was performed stereotaxically at anteroposterior (AP) −3.1 mm and mediolateral (ML) −1.2 mm relative to the bregma and dorsoventral (DV) −4.0 mm from the dural surface in three‐month‐old C57BL/6J mice. A total of 300 nL of viral suspension was injected into each site at a speed of 40 nL/min with a 10 μL glass syringe with a fixed needle. The needle was left in place for an additional 5 min before it was removed slowly.

### 4.20 Mass spectrometry analysis

LC‐MS/MS identification and data analysis were performed by SpecAlly Life Technology (SpecAlly Life Technology Co., Ltd.) as previously described (Zhou et al., 2022). In brief, the target protein bands in the gel were cut into pieces, washed three times with 50% acetonitrile/100 mM NH_4_HCO_3_, and digested in 50 mM NH_4_HCO_3_ solution (pH 8.0) with MS‐grade trypsin overnight at 37°C after reduction and alkylation of cysteines. The tryptic digests were injected into an Easy‐nLC 1200 system (Thermo Scientific) and analyzed by a Q Exactive plus mass spectrometer (Thermo Scientific). The original files were analyzed using the Sequest HT search algorithm and Proteome Discoverer (version 2.4). The MS1 match tolerance was set at 10 ppm; the MS2 tolerance was set at 0.02 Da. The minimum peptide length was set to 6 amino acids, and a maximum of three miscleavages was allowed. We filtered the search results with 1% FDR at the protein and peptide levels.

### 4.21 Generation of the K182Hcy antibody

To generate the K182Hcy antibody, the synthesized peptide AAQVKAPLV containing K182 N‐hcy was used as the antigen to immunize rabbits. The antibody was produced by Abmart Shanghai Co., Ltd. Antiserum was collected after five sessions of immunization. The titers against the immunizing peptide were determined by ELISA. The antiserum was purified by affinity chromatography and then counterscreened with the peptide AAQVKAPLV without N‐hcy.

### 4.22 Chemoselective labeling of N‐homocysteinylated DJ‐1

The reactions were performed as previously reported (Chen et al., 2019). The reaction solutions were labeled with biotin‐azide (200 μM final concentration; MedChemExpress, HY‐129832). Freshly made hemin (50 μM final concentration), β‐mercaptoethanol (100 mM final concentration), and SDS (0.4% final concentration) were added together. The mixture was heated at 75°C for 10 min. Then, the samples were heated at 95°C for 10 min with 5× loading buffer, followed by 10% Bis‐Tris SDS‐PAGE.

### 4.23 Statistical analyses

All data were expressed as means ± SEM (standard error of the mean) from three or more independent experiments and illustrated with GraphPad Prism GraphPad 9.2.0 (GraphPad Software Inc., San Diego, CA, United States). *p* < 0.05 was considered to be statistically significant. One‐way ANOVA was applied to confirm the significant main effects and differences among three or more groups followed by Tukey's post hoc multiple comparisons. Kruskal–Wallis and Dunn's multiple comparisons were used to analyze the distributions among groups. All experiments were performed in triplicate for at least three independent trials.

## AUTHOR CONTRIBUTIONS

Z.Z. conceived and supervised the project. T.G. performed most of the experiments and wrote the draft. Z.L. performed the chemoselective labeling and helped in designing the methodology. M.X., J.X., J.H., Y.L., and G.Z. helped with the cellular and animal experiments. G.C., Z.W., T.X., D.H., and A.B. helped in the data analysis.

## CONFLICT OF INTEREST STATEMENT

The authors declare that no conflicts of interest exist.

## Supporting information


Appendix S1.

## Data Availability

All data generated or analyzed in this study are available in the published article.
