# Supplementary material for: N‐homocysteinylation of DJ‐1 promotes neurodegeneration in Parkinson's disease
Source: Aging Cell. 2024 Feb 21;23(5):e14124. doi: 10.1111/acel.14124 (PMC11113254; doi:10.1111/acel.14124)
Supplement: Supplementary file 1 — Appendix S1. [file ACEL-23-e14124-s001.docx]

**
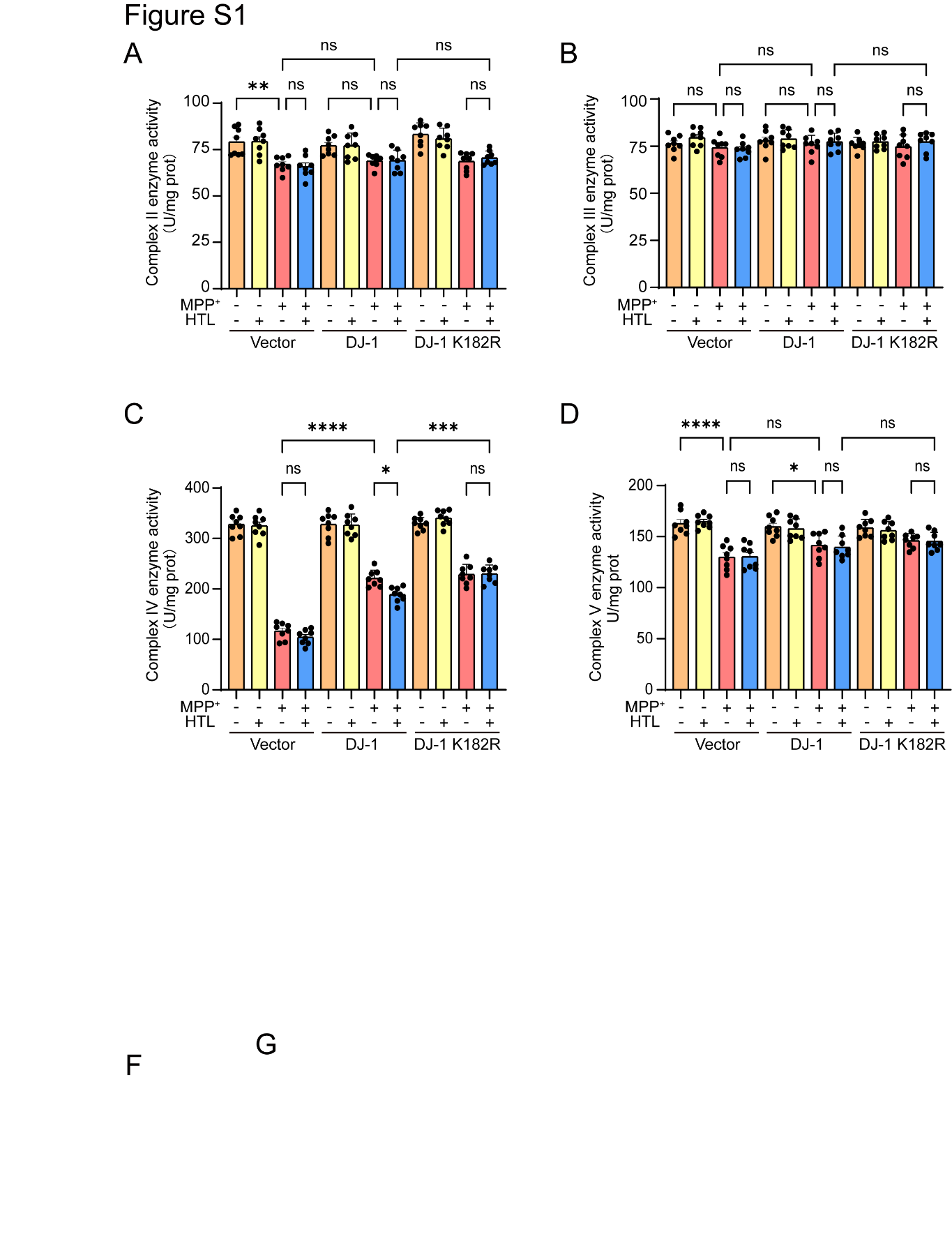
**

**Supplementary Figure 1. Complex II-V enzymatic activity of mitochondria from SH-SY5Y cells.** SH-SY5Y cells expressing wild-type or K182R mutant DJ-1 were exposed to MPP^+^ and HTL. The enzymatic activity of mitochondrial Complex II-V was detected. Data are shown as mean ± SEM. n = 8 independent experiments. *P* values were determined by one-way ANOVA followed by Tukey’s multiple comparisons. **P* < 0.05, ***P* < 0.01, ****P* < 0.001, *****P* < 0.0001, ns, not significant.

**
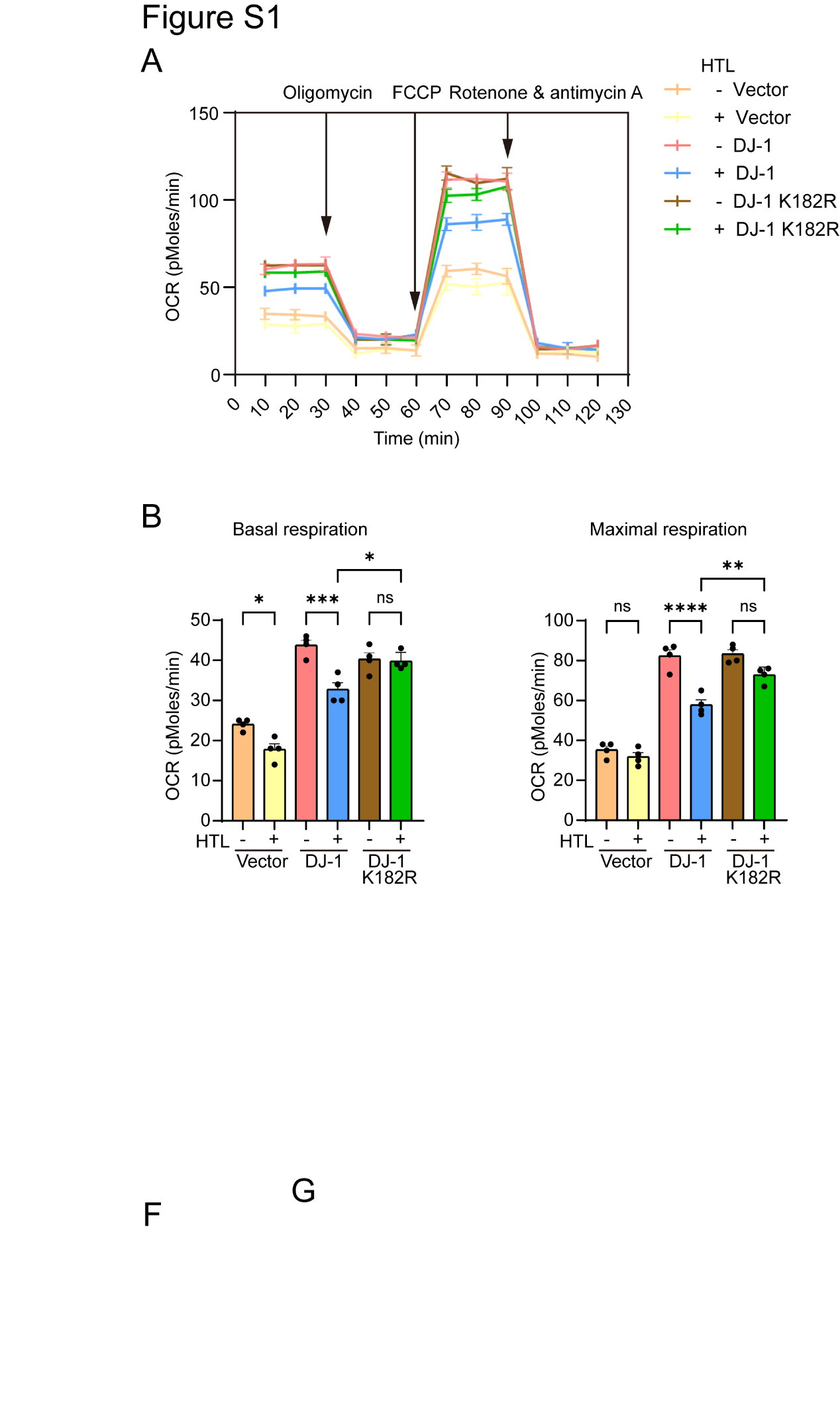
**

**Supplementary Figure 2. Oxygen consumption rate (OCR)** **assay of mitochondria from SH-SY5Y cells.** SH-SY5Y cells expressing wild-type or K182R mutant DJ-1 were exposed to MPP^+^ and HTL. **(**A) The OCR of mitochondrial was detected. **(**B) Bar diagrams representing basal respiration and maximal respiration from the data obtained from (A). Data are shown as mean ± SEM. n = 4 independent experiments. *P* values were determined by one-way ANOVA followed by Tukey’s multiple comparisons. **P* < 0.05, ***P* < 0.01, ****P* < 0.001, *****P* < 0.0001, ns, not significant.


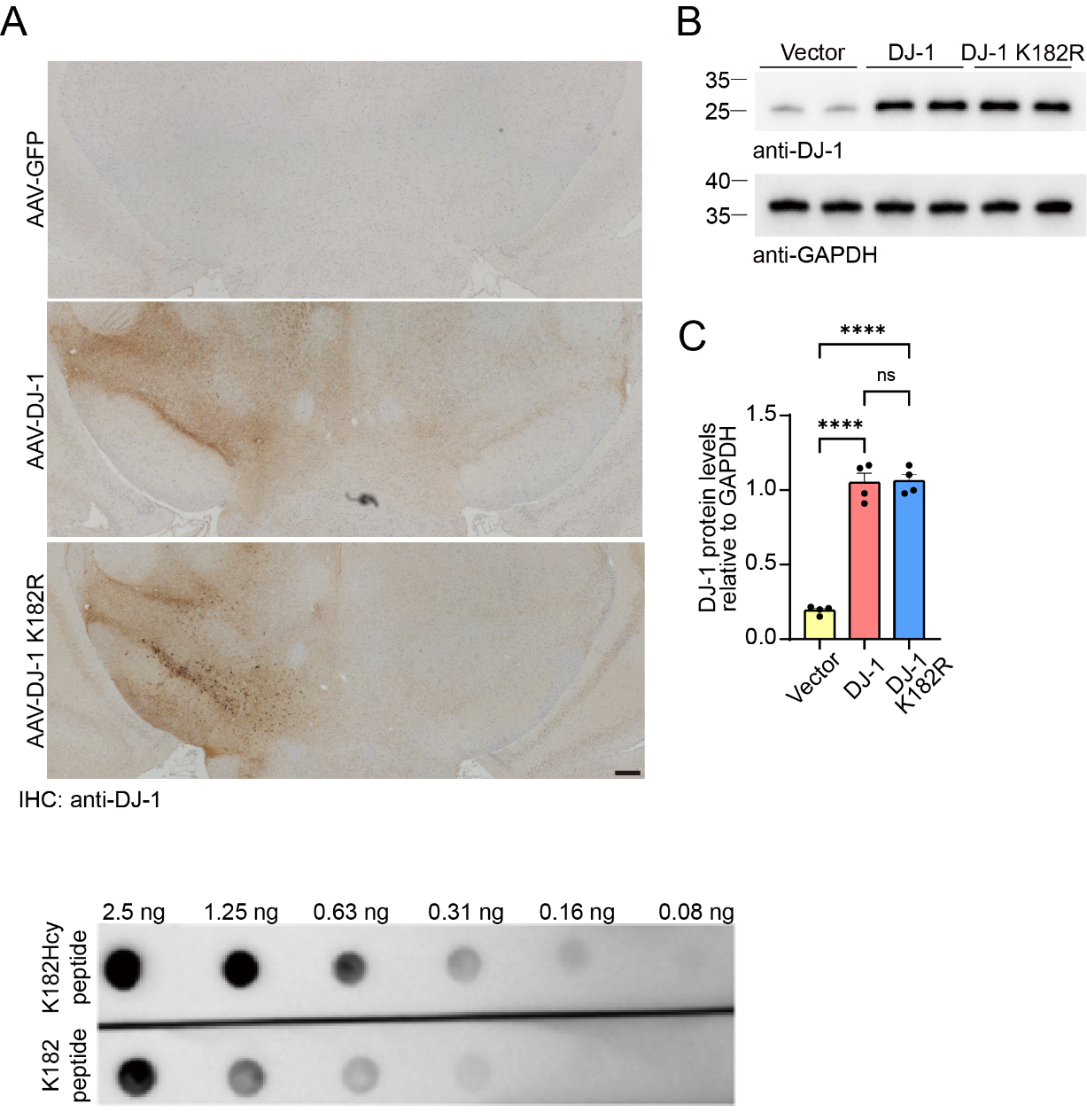


**Supplementary Figure 3. Verification of DJ-1 expression in the brains of AAV-injected mice.**

Adeno-associated viruses (AAVs) were injected into the substantia nigra (SN) of three-month-old WT mice. (**A**) Immunohistochemistry (IHC) of DJ-1 in the SN. (**B**) Levels of DJ-1 in the ipsilateral SN. (**C**) Quantification of DJ-1. Results were normalized to GAPDH. n = 4 mice per group. *P* values were determined by one-way ANOVA followed by Tukey’s multiple comparisons. *****P* < 0.0001, ns, not significant. Scale bar = 200 μm.


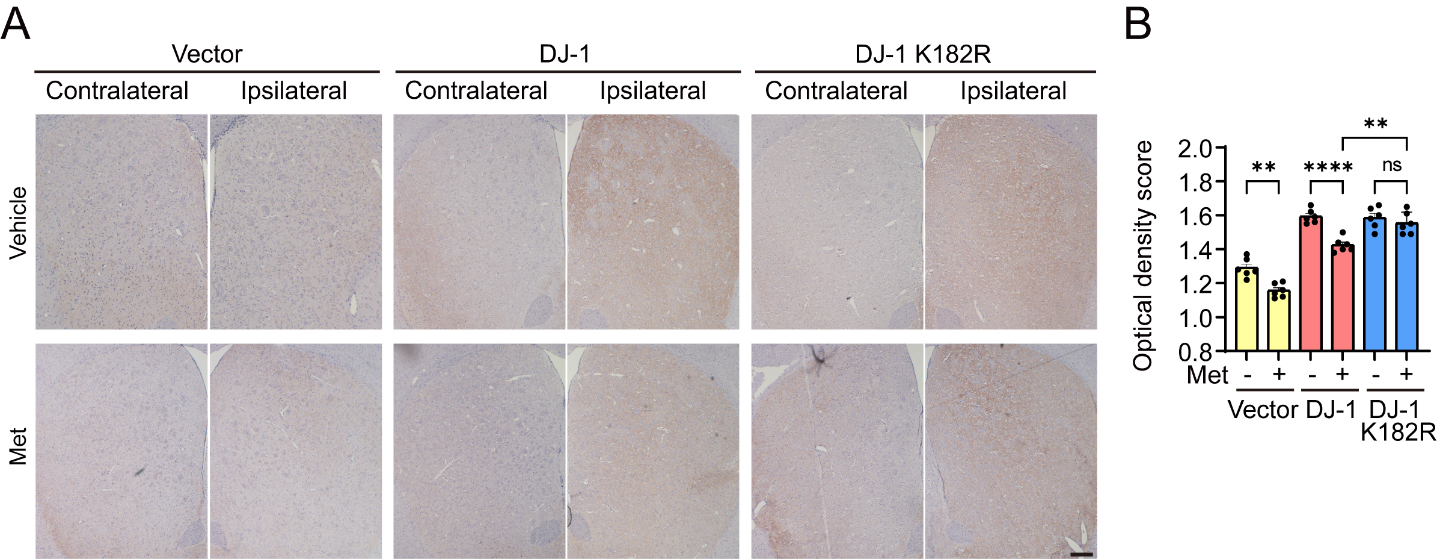


**Supplementary Figure 4. Blockade of DJ-1 K182Hcy attenuates** **the decrease of TH levels in the striatum induced by Hcy.**

(**A**) TH immunohistochemistry images in the striatum. (**B**) Quantitation of average optical densities of dopaminergic terminals in the ipsilateral striatum. n = 6 mice per group. *P* values were determined by one-way ANOVA followed by Tukey’s multiple comparisons. ***P* < 0.01, *****P* < 0.0001, ns, not significant. Scale bar = 200 μm.

**Table S1. Homocysteinylated lysine residues on DJ-1 detected by mass spectrometry.**

| Peptide position  (DJ-1) | Peptide Sequence^1^ | Position in peptide | Ratio mod/base | Intensity |
| --- | --- | --- | --- | --- |
| 176-189 | EVAAQVKAPLVLKD | 7 | 9.8941 | 4513200 |
| 49-63 | DVVICPDASLEDAKK | 14 | 0.042651 | 2768700 |
| 123-132 | VTTHPLAKDK | 8 | 3.8512 | 1697500 |
| 33-48 | VTVAGLAGKDPVQCSR | 9 | 0.025587 | 1366200 |
| 90-99 | EILKEQENRK | 4 | 0.068544 | 1353500 |
| 29-48 | AGIKVTVAGLAGKDPVQCSR | 4 | 0.17114 | 842480 |
| 6-27 | ALVILAKGAEEMETVIPVDVMR | 7 | 1.5782 | 841850 |
| 64-93 | EGPYDVVVLPGGNLGAQNLSESAAVKEILK | 26 | 0.35926 | 725050 |
| 99-122 | KGLIAAICAGPTALLAHEIGFGSK | 1 | 0.017675 | 621560 |
| 63-89 | KEGPYDVVVLPGGNLGAQNLSESAAVK | 1 | 0.0042463 | 380510 |
| 146-156 | VEKDGLILTSR | 3 | 0.031303 | 236540 |

HEK293 cells expressing HA-tagged DJ-1 were exposed to 0.1 mM HTL for 24 h. DJ-1 was purified using HA beads and subjected to mass spectrometry (MS). Homocysteinylated lysine residues are highlighted.

**Table S2. DJ-1 peptides detected by mass spectrometry.**

| Sequence | Length | Modification | Modified Position^1^ | Intensity |
| --- | --- | --- | --- | --- |
| EVAAQVKAPLVLKD | 14 | Hcy | EVAAQVKAPLVLKD | 122410 |
| EVAAQVKAPLVLKD | 14 | Unmodified |  | 84365 |
| DVVICPDASLEDAKK | 15 | Unmodified |  | 61209 |
| GAEEMETVIPVDVMR | 15 | Oxidation | GAEEMETVIPVDVMR | 55752 |
| GAEEMETVIPVDVMRR | 16 | Oxidation | GAEEMETVIPVDVMRR | 48404 |
| GAEEMETVIPVDVMRR | 16 | Unmodified |  | 45566 |
| DVVICPDASLEDAKK | 15 | Hcy | DVVICPDASLEDAKK | 45288 |
| GAEEMETVIPVDVMR | 15 | 2 Oxidation | GAEEMETVIPVDVMR | 44980 |
| EGPYDVVVLPGGNLGAQ  NLSESAAVK | 26 | Unmodified |  | 34172 |
| DGLILTSR | 8 | Unmodified |  | 19317 |
| AGIKVTVAGLAGKDPVQ  CSR | 20 | Unmodified |  | 19291 |
| GAEEMETVIPVDVMR | 15 | Unmodified |  | 18130 |
| KEGPYDVVVLPGGNLGA  QNLSESAAVK | 27 | Hcy | KEGPYDVVVLPGGNLGA  QNLSESAAVK | 14280 |
| ALVILAK | 7 | Unmodified |  | 10452 |

HEK293 cells expressing HA-tagged DJ-1 were exposed to 0.1 mM HTL for 24 h. DJ-1 was purified using anti-K182Hcy antibody and subjected to mass spectrometry (MS). Homocysteinylated lysine residues are highlighted.
